# Material Properties Changes Caused by High Temperature Drying—Corn Cobs Case Study

**DOI:** 10.3390/ma18102302

**Published:** 2025-05-15

**Authors:** Marek Wróbel, Marcin Jewiarz, Jozef Krilek, Luiza Dmochowska-Kuc

**Affiliations:** 1 Department of Mechanical Engineering and Agrophysics, University of Agriculture in Krakow, Balicka 120, 31-120 Kraków, Poland; marcin.jewiarz@urk.edu.pl (M.J.); luizadmoch@gmail.com (L.D.-K.); 2 Department of Environmental and Forestry Machinery, Faculty of Technology, Technical University in Zvolen, T.G. Masaryka 24, 960 01 Zvolen, Slovakia; jozef.krilek@tuzvo.sk

**Keywords:** biomass drying, grindability, pellets, solid density, mechanical durability

## Abstract

Biomass is an energy source with variable physico-chemical properties. Thermal treatments lower moisture and volatile matter contents. They also raise the high heating value (HHV). This is especially desirable for agro-wastes with low-energy potential, like maize cobs. To make pellets from biomass, it is important to keep the lignin intact. It is responsible for particle adhesion. This paper presents a study focused on high-temperature drying of maize cobs. The process temperatures were selected from a range between 60 and 220 °C. The upper temperature limit prevents significant lignin breakdown. We also do not exceed the self-ignition temperature of the raw material. The study analyzed changes in basic technical parameters. These include moisture content, ash content, volatile matter, and HHV. We tested the grinding and densification process. We measured the raw material’s particle size distribution (PSD), specific density, and the mechanical durability (DU) of the agglomerates. The study showed a positive effect of high-temperature drying on the technical parameters. We found that the drying of corn cobs at a temperature of 180 °C gives the best results. Both PSD and DU values indicate that it is possible to create quality compacted biofuels from this material.

## 1. Introduction

Biomass, as renewable organic matter source, holds significant promise for sustainable energy production and various industrial applications. Thermal treatment methods, including drying, torrefaction, pyrolysis, and gasification, are employed to enhance biomass quality. These processes convert biomass into energy-dense intermediates or final products with broader applications. The thermal processes can induce significant changes in the physical and chemical structure of biomass. This leads to a change in its properties and suitability for specific applications.

### 1.1. Biomass Composition and Structure

Biomass primarily comprises lignocellulosic materials: cellulose, hemicellulose, and lignin [1]. Cellulose is a crystalline polysaccharide that provides structural support. It is relatively stable at lower temperatures, but high temperatures can induce significant structural changes [2]. Some studies have shown that high temperatures can lead to a decrease in the degree of polymerization (DP) of cellulose [3]. This means the long chains of glucose molecules that make up cellulose are broken down into shorter segments [4].

Hemicellulose, an amorphous heteropolymer, is more easily degraded than cellulose. We can observe a significant degradation event during high-temperature drying. This degradation typically begins at temperatures above 150 °C, leading to the release of volatile organic compounds [5] and a reduction in the biomass’s overall mass [6]. The removal of hemicellulose can increase the relative proportion of cellulose and lignin in the remaining solid material. This can improve the biomass’s heating value and its suitability for thermochemical conversion processes.

Lignin, being a complex and robust polymer, exhibits higher thermal stability compared to cellulose and hemicellulose [2,3]. During pyrolysis, lignin breaks down into a complex mixture of phenolic compounds, contributing to the bio-oil fraction and the formation of char [1]. The thermal behavior of lignin is also influenced by its hydration and thermal history, with a dynamical and structural hysteresis observed at different temperatures [7].

### 1.2. Thermal Treatments of Biomass

Thermal treatments in general are processes requiring the use of elevated temperatures. In the case of biomass, we can distinguish four main processes:

- Drying;
- Pyrolysis, including torrefaction;
- Gasification;
- Combustion.

Combustion, as the most extreme example, destroys lignocellulosic structures. Therefore, it cannot be considered an upgrading.

Drying is a set of technological operations aimed at reducing the water content of biomass. Evaporation is the most common method of water removal from biomass. By increasing the temperature or reducing the ambient pressure, evaporation can be intensified. For solid fuels, including biomass, thermal drying is the most common method. Reducing the moisture content not only affects the energy parameters (by increasing the calorific value) but also reduces the intensity of biological decomposition.

Pyrolysis involves heating biomass in the absence of oxygen at temperatures typically between 300 and 900 °C. This process breaks down the biomass into solid (biochar), liquid (bio-oil), and gaseous products, with the relative yields depending on the operating conditions and biomass type [1,8]. The torrefaction is a mild version of the pyrolysis process conducted at temperatures ranging from 200 to 300 °C [9,10]. It aims to improve the fuel properties of biomass by removing moisture and volatile organic compounds. This results in a more homogeneous, energy-dense, and hydrophobic product [5].

Gasification is a high-temperature process (typically above 700 °C) under sub-stoichiometric presence of oxygen. It converts biomass into a gaseous mixture, primarily composed of carbon monoxide, hydrogen, and methane [11]. This gas can be used for power generation, heat production, or as a feedstock for chemical synthesis.

### 1.3. Changes of Biomass During Thermal Treatments

Thermal treatment significantly alters the chemical composition of biomass. Torrefaction primarily decomposes hemicellulose, leading to a reduction in the O/C and H/C ratios [12]. This increases the relative concentration of lignin and fixed carbon, enhancing the energy density of the treated biomass [13]. Pyrolysis and gasification cause more extensive decomposition of all three major components (cellulose, hemicellulose, and lignin), resulting in a carbon-rich solid residue (biochar) and volatile products [1].

Thermal treatment also induces changes in the physical properties of biomass, such as grindability, porosity, and surface area [14,15]. Torrefaction improves the grindability of biomass by weakening the cell wall structure, making it easier and less energy-intensive to mill. Pyrolysis can create a porous structure in biochar, increasing its surface area and making it suitable for applications like adsorption and catalysis [5,16].

Drying influences the physico-chemical quality of biomass. Process temperature significantly influences the rate of moisture removal and induces various physical and chemical transformations within the biomass. A study by Cai et al. [17] demonstrated a parabolic relationship between critical moisture content and drying temperature, with critical moisture content decreasing as temperature increased. This indicates that higher temperatures accelerate the drying process, but the relationship is not linear. The researchers also found that drying temperature significantly affected effective diffusivity and the mass transfer coefficient during the dramatically falling period of biomass drying [17]. Conversely, during the slowly falling period, the effect was less pronounced. These findings underscore the importance of considering the drying stage when optimizing process parameters. The effect of temperature extends beyond moisture removal. Gustafson and Morey [18] explored the impact of drying air temperature on grain quality parameters, observing effects on test weight, kernel volume, true density, and breakage susceptibility. This suggests that high-temperature drying, while efficient for moisture removal, may compromise certain quality attributes depending on the biomass type and desired end-use.

High-temperature drying induces various chemical transformations within the biomass. The degradation of hemicellulose and other components is frequently observed [19]. The formation of new chemical bonds, such as cross-linking structures, can also occur [20]. The Maillard reaction, a non-enzymatic browning reaction, is often observed in high-temperature drying, leading to color changes and potentially affecting the nutritional value of the biomass [21,22]. Lipid oxidation can also occur, leading to the formation of undesirable compounds. The type and extent of these chemical transformations depend on the biomass composition, drying temperature, and drying method. The generation of volatile organic compounds (VOCs) during the drying process is also a potential concern [22,23]. The composition of these VOCs can vary depending on the biomass type and drying conditions.

The impact of high temperatures on the color of the dried biomass has also been studied. Klement and Marko [24] investigated color changes in beech wood during high-temperature drying (HT drying), finding that darkening depended on temperature, moisture content, drying time, and sample density. Möttönen and Kärki [25] similarly studied birchwood, noting significant darkening during high-temperature drying if steam was used as the drying medium. These studies highlight the importance of carefully controlling drying parameters to achieve desired color characteristics in the final product. Liang et al. [21] designed a bidirectional alternating hot air drying system to mitigate browning reactions in thick-layered biomass, demonstrating that drying efficiency and uniformity can be improved by controlling airflow direction and temperature.

### 1.4. Importance of Drying in Pellets Production

Moisture content plays a vital role in the pelleting process by influencing the binding characteristics of the material [26,27,28]. Insufficient moisture can lead to poor binding, resulting in low durability and the generation of excessive fines (small particles) [29]. Conversely, excessive moisture can also reduce pellet quality by causing swelling and disintegration during storage, as well as increasing the risk of microbial growth. Therefore, maintaining an optimal moisture range is critical for producing durable pellets.

The effect of moisture content on pellet durability is also linked to the type of biomass used. For instance, woody biomass and non-woody biomass have different optimal moisture ranges for pelleting [30]. Generally, such a stream of biomass requires more careful moisture management due to its diverse composition and structural properties [31].

Mechanical durability refers to the pellet’s ability to withstand handling and transportation without breaking or producing excessive fines. Optimal moisture content enhances the binding between particles, resulting in higher durability. Conversely, excessive moisture can weaken the pellet structure, leading to reduced durability [32].

Drying temperature can affect the quality of non-woody biomass. Some research papers highlight its significant impact on structural changes, chemical composition, and processing characteristics. Elevated drying temperatures can alter the biomass’s cellular structure, reducing moisture content while affecting hemicellulose degradation and lignin softening, which influences grindability. Proximate and ultimate analyses reveal that higher temperatures lead to increased fixed carbon and reduced volatile matter, impacting combustion efficiency. Moreover, drying temperature affects pellet production by modifying particle binding properties, where excessive heat can degrade natural binders, leading to poor pellet durability. Optimizing drying conditions is crucial to balancing energy efficiency, mechanical properties, and fuel quality of non-woody biomass for industrial applications.

### 1.5. Grinding

Grinding is a fundamental step in pellet production. It plays a critical role in determining the quality and characteristics of the final product. The process of grinding reduces the size of raw materials. An increase in the surface area results in better binding during pelleting. Grinding affects pellet quality [14]. Powder characteristics such as shape and particle size distribution influence pellet quality [16]. The choice of grinding method affects the pellet quality [15]. Determining the optimal drying conditions for effective grinding depends on various factors, including biomass type, initial moisture content, and intended application. For brewers’ spent grains (BSGs), from an operational standpoint, research indicates that the most suitable temperature for the drying process is 105 °C, as it allows for shorter drying times without markedly affecting the total phenolic content [2].

The choice of drying method also influences grinding effectiveness. In a study on novel drying methods for upcycling (BSG) as a plant protein source, researchers explored the dehydration kinetics of BSG and the effect of three different drying methods—oven drying (OD), freeze drying (FD), and vacuum microwave drying (VMD)—on their protein content and functionality. The vacuum microwave drying (VMD) process took less drying time (48 min) compared to oven drying (50 min), with higher effectiveness as a drying process. VMD-treated BSG also showed moderate protein functionality and the highest overall acceptability when used in baked chips, suggesting that VMD might be used as a sustainable drying technology for thermal treatment and valorization of BSG [16]. Literature analysis shows deficiencies in the area of comprehensive analysis of the waste biomass grinding process.

## 2. Materials and Methods

### 2.1. Material

**Figure 1:**
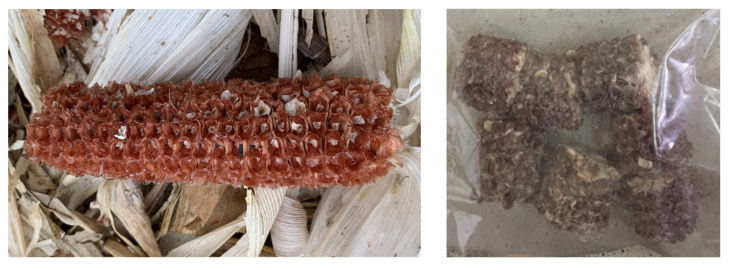
Testing material: (**left**)—after harvesting, (**right**)—prepared for drying.

The test material was maize cob cores (Figure 1) of the *Keltikus* variety. This is the residue from harvesting this crop for grain. It is a fodder variety. Its main use is as a grain crop for fodder or bioethanol. All material was collected after harvest in autumn 2024. Before drying, the material was cut into fragments approximately 20 mm long.

### 2.2. Drying

We divided a sample of corn cob biomass into five subsamples. Then, we dried them at temperatures of 60, 100, 140, 180, and 220 °C for 24 h. The process was conducted in a laboratory dryer (SLW 115, Pol-Eko, Wodzisław Śląski, Poland). The aim of the studies was to find out how drying affects material quality. This included energy parameters like ash content, volatile matter, and high heating value. Further, we made grindability, compressibility, and compactability analyses. To avoid the effects of moisture content, we made all analyses on the material in a dry state.

The drying temperature was set below the relative self-ignition temperature (RSIT) of the test material. As a result, the drying process did not require the use of a protective atmosphere. The RSIT of corn cobs was measured according to the guidelines contained in EC No 440/2008 [33]. That document defines the method and equipment to determine the RSIT of solids. We used a test stand built in accordance with this guideline by Czylok company (Jastrzębie-Zdrój, Poland). Detailed description of this procedure is presented in the previous publications [34]. According to it, the RSIT of the tested corn cobs was 227.6 °C.

### 2.3. Energy Parameters

Ash content (*A*), volatile matter content (*VM*), and net calorific value $q_{p,net}$ were determined according to solid biofuel standards (respectively: ISO 18122:2022 [35], ISO 18123:2023 [36], and ISO 18125:2017 [37]). The material was prepared according to ISO 14780:2017 [38], i.e., the sample was milled on a laboratory grinder, achieving a grain size of less than 1 mm.

### 2.4. Grinding

The material was ground using the method described in paper [15]. This stage was divided into pre-grinding and basic grinding. The goal was to collect uniform samples for particle size distribution (PSD) during the basic grinding stage.

In the first stage, we ground the dried and chopped cobs using a knife milling system (Testchem LMN-100, Pszów, Poland). It was equipped with a 6 mm sieve. We analyzed samples of ground material for their particle size distribution based on EN ISO 17827-2:2024 [39]. A sieve set with hole sizes 6; 3.15; 2; 1.4 1; 0.5; 0.25 mm; and an LPzE-4e shaker from Morek Multiserw, Marcyporęba, Poland, have been used for this purpose. As a result, the tested samples were divided into eight sieve classes B1–B8 (Table 1).

**Figure 2:**
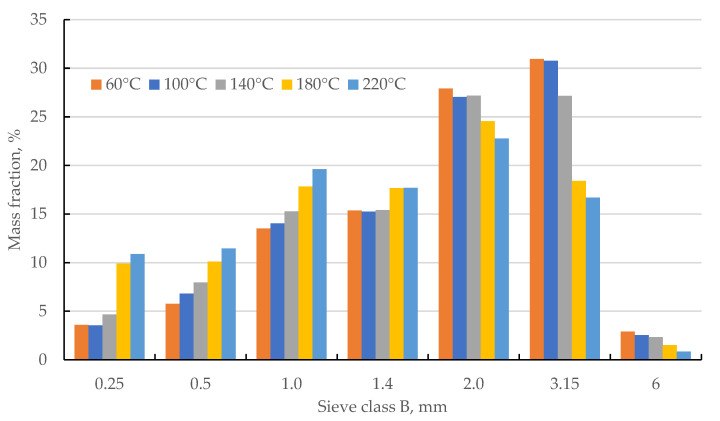
The samples’ particle size distribution after the preliminary grinding stage.

The PSD samples after preliminary grinding varied (Figure 2). This could have been caused by the drying temperature but also by the difference in the size of the cob pieces obtained before drying. Therefore, before the main grinding stage, the PSD of all samples was standardized to avoid the influence of different input PSD of the sample on its output PSD.

The standardized PSD included only fractions B_3_, B_4_, and B_5_ mixed in the proportions listed in Table 2. So, the prepared samples were in the main stage ground in a hammer mill (PX-MFC 90D Polymix, Kinematika, Luzern, Switzerland) with a 2 mm sieve installed.

After the second grinding samples were divided into 7 C sieve classes (Table 3) to determine PSD. Also, the cumulative PSD and the median value of particle size *d*_50_ were determined.

### 2.5. Compressibility and Compactability

The ground material was used to determine the course of the compaction process. Single portions of material (0.7 g) were compacted on a closed channel stand. The mass of the sample was selected so that the height of the pellet was about 0.5 of its diameter. The main parts of the stand are a cylindrical chamber (length of 110 mm and internal diameter of 12 mm), a piston, and a bottom counter-piston (diameter of both—11.9 mm). All these parts are made of hardened steel. The compaction pressure was selected based on the results of the research team’s previous studies conducted on a wide range of biomass types. Based on these data, the tests were carried out at 457.8 MPa.

The movement of the piston was induced by the head of the testing machine, Wance TestStar (TSE255D, Shenzhen Wance Testing Machine Co., Ltd., Shenzhen, China). The piston movement was continued until the specified compaction pressure was reached. When the value of 457.8 MPa was achieved, the piston stopped and remained in this position for 60 s. After this time, the pressure on the piston was released and the counter piston was removed. The repeated piston move pushed the pellet out of the chamber.

The obtained agglomerates were placed in string bags and stored for 24 h. After this time, the compressibility and compactibility of the test material were measured. Compressibility of a granular material is its ability to decrease in volume, and thus increase in density, as a result of the pressure exerted on the material. Compactibility, on the other hand, is the ability of a material to form a mechanically strong granule as a result of the pressure exerted on that material. The measure of compressibility is the density of the resulting pellets, and the measure of compactibility is their mechanical strength.

Based on the measurement of the geometry of the obtained pellets and their weight, the specific density of DE pellets was calculated from the formula:

$$DE=\frac{m}{V}$$

where

*DE*—specific density of the pellet (g/cm^3^),

*m*—mass of the pellet (g),

*V*—volume of the pellet (cm^3^).

The mechanical strength of the pellets should be realized according to the guidelines of EN ISO 17831-1 [40]. However, this standard requires a 500 g pellet sample per test. The mass of pellets produced for a given drying temperature was much smaller compared to that required by this standard. The procedure and apparatus for determining the mechanical strength of tablets (Tablet friability) contained in the U.S. Pharmacopeia [41], also used in the European and Japanese Pharmacopoeia, was used. A sample of 10 whole tablets should be carefully de-dusted prior to testing. Then, the tablets were weighed and placed in the special drum. The drum rotates 100 times at a speed of 25 ± 1 rpm. After testing, any loose dust from the tablets is removed as before, and the tablets are accurately weighed. A weight loss of no more than 1.0% is considered acceptable for most products. In the present study, the pellets were weighed after 50 and 100 revolutions and the measure of the mechanical strength of the pellets DU was not the percentage weight loss, but the percentage of pellets that did not crumble (this is the way of representing the mechanical strength of pellets is used in PN-EN ISO 17831-1. DU was determined from the relationship:

$$DU=\frac{m_{A}}{m_{E}}\cdot 100$$

where

*DU*—sample durability (%),

*m_A_*—sample mass before test (g),

*m_E_*—sample mass after test (g).

## 3. Results

### 3.1. Energy Parameters

**Figure 3:**
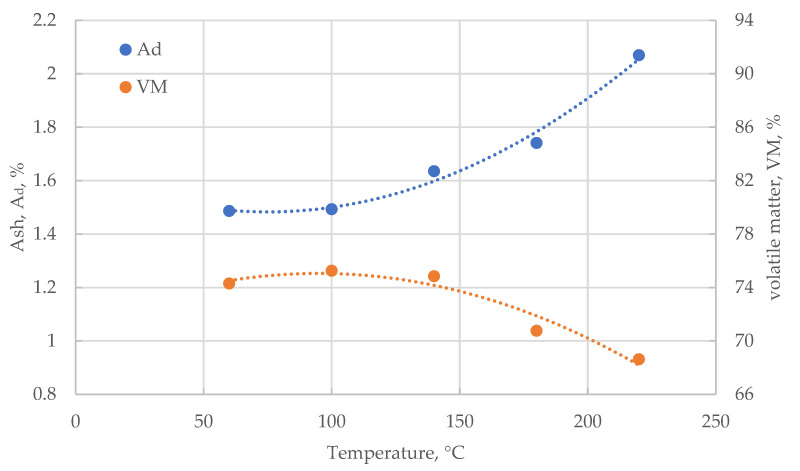
Changes in ash and volatile matter content of corn cobs samples with drying temperature.

The ash content ($A_{d}$) of the various dried samples are shown in Figure 3. The drying temperature affects the material parameters. The ash content of the material dried at 60 and 100 °C remains at a similar level of 1.5%. Comparing this to the requirements of the wood pellet quality standard (ISO 17225-6:2021 [42]), it turns out that the tested corn cob biomass does not meet the requirements of class A1 and A2 in this case (which require $A_{d}$ ≤ 0.7% and $A_{d}$ ≤ 1.2%, respectively); however, it already meets the requirements of class B ($A_{d}$ ≤ 2.0%). Of course, the tested biomass is not woody biomass and we cannot require it to fully meet the high requirements for woody biomass. The obtained results are within a certain range of these requirements. Comparing the results to the requirements of the quality standard for non-wood pellets (ISO 17225-2:2021 [43]) and, therefore, pellets produced from, for example, corn cob, it turns out that the obtained ash compactness is significantly lower than the required minimums (Class A: $A_{d}$ ≤ 6.0%; Class B: $A_{d}$ ≤ 10.0%).

Corn cob is, in terms of ash content, a good raw material for non-wood pellet production. An increase in drying temperature to 140 °C is associated with an increase in Ad content to only 1.6%, and a drying temperature of 180 °C increases Ad to 1.7%. Thus, it is still biomass Class B wood pellets. Only a temperature of 220 °C (according to ISO 17225-1:2021 [44], it is already a thermally treated biomass fuel) increases Ad to a level of 2.1%. It no longer meets the requirements of ISO 17225-2:2021; however, it is still within the requirements of ISO 17225-6:2021. In the temperature range tested, we observe an increase in ash content, which is an undesirable phenomenon, but this increase does not disqualify corn cobs as a raw material for non-wood pellet production.

The volatile matter content in a dry state ($VM_{d}$) analysis shows an opposite trend to the $A_{d}$ analysis (Figure 3). The $VM_{d}$ content of the dried material in the temperature range of 60–140 °C is practically the same as 74.5%. The temperature of 180 °C causes a decrease in $VM_{d}$ to 70.8% and the temperature of 220 °C to 68.6%. The decrease in the content of volatile parts is a desirable phenomenon; so, in this case, the higher the drying temperature, the better the material’s parameters in terms of $VM_{d}$.

**Figure 4:**
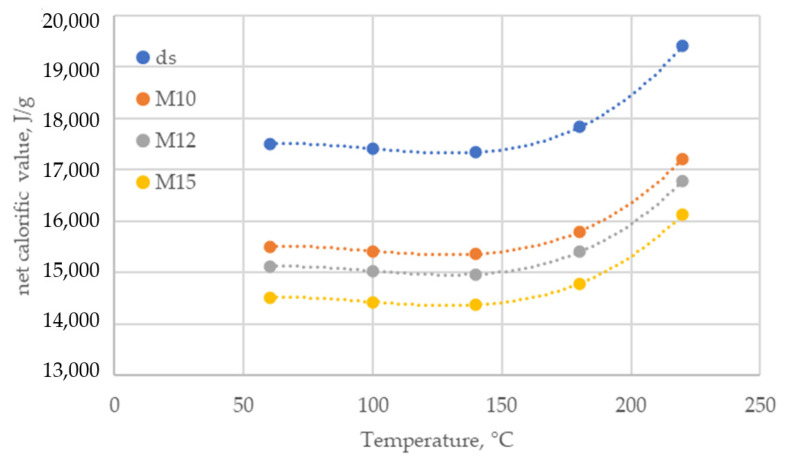
Changes in net calorific value of corn cobs samples with drying temperature.

As for the most important energy parameter (net calorific value in the dry state ($q_{p,net,d}$)), the course of change induced by drying temperature is clear (Figure 4—curve “ds”). In the range of 60–140 °C, the net calorific value $q_{p,net,d}$ decreases from 17,510 J/g to 17,340 J/g, and this is, of course, a slight decrease. An increase in temperature to 180 °C already causes an increase in $q_{p,net,d}$ to 17,830 J/g. On the other hand, a drying temperature of 220 °C causes the resulting material to reach $q_{p,net,d}$ = 19,410 J/g. From the point of view of the combustion process, corn cobs should be dried at 220 °C because then the obtained $q_{p,net,d}$ is the highest. Relating the obtained results to ISO 17225-2:2021 and ISO 17225-6:2021 standards, the obtained value of $q_{p,net,d}$ meets the required minima—$q_{p,net}$ ≥ 16.5 MJ/kg and $q_{p,net}$ ≥ 14.5 MJ/kg, respectively—regardless of the drying temperature. However, it should be noted that these are $q_{p,net}$ values for the dry state. According to ISO 17225-2:2021, the permissible maximum moisture content ($M_{ar})$ of pellets of all quality classes is 10%. However, according to ISO 17225-6:2021, for class A, the moisture content can be at 12%, and for class B, it can be at 15%. When we take into account the maximum moisture level of material $M_{ar}$, after converting the results from the dry state to the working state, the value of $q_{p,net}$ will decrease accordingly (Figure 4—curves M10, M12, and M15).

Analyzing the curves of net calorific value in the as-received state ($q_{p,net,ar}$), it was found that the obtained value of this parameter does not always reach the required minimum. The requirements of ISO 17225-2:2021 ($M_{ar}$ = 10%, $q_{p,net,ar}$ ≥ 16.5 MJ/kg) are met only by material dried at 220 °C. In the case of the requirements of ISO 17225-6, $M_{ar}$ = 12% (the acceptable level of $M_{ar}$ class A), and the values of $q_{p,net,ar}$ are above the required minimum of 14.5 MJ/kg, regardless of the drying temperature. However, if we increase the moisture content of the material to 15% (permissible level $M_{ar}$ of class B), then only the $q_{p,net,ar}$ values of the material dried at 60, 180, and 220 °C meet the required minimums.

It is possible to reduce the moisture content of the material accordingly and get into the range of the required values of $q_{p,net}$, but often the pressure compaction process requires material at a moisture content level of 8–10% and even higher [45] because then the process runs properly. After the compaction process, the finished pellet can be dried to the required moisture level so that $q_{p,net}$ is in accordance with the requirements of the standard, but this dependence of $M_{ar}$ on $q_{p,net}$ should be taken into account in the technological process.

### 3.2. Grindability in Aspect of Particle Size Distribution Changes

**Figure 5:**
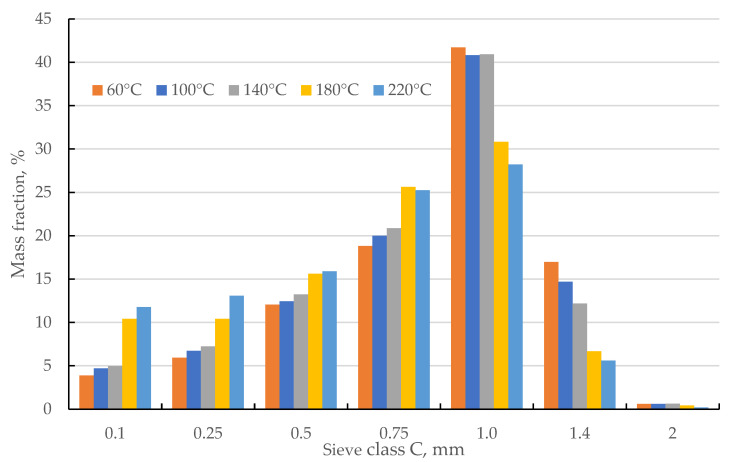
The samples’ particle size distribution (PSD) after the main grinding stage.

The particle size distribution (PSD) after final grinding also varied with drying temperature (Figure 5). The course of PSD changes is similar to that of pre-grinding, even though the ground samples had the same PSD at the input. This means that the drying temperature levels studied change the structure of the material. This affects the course of the grinding process. Similar relationships have been observed in earlier studies by other authors [46] and our own research [15]. For temperature levels of 60, 100, and 140 °C, the proportion of C:0.1, 0.25, 0.5, and 0.75 fractions increases with increasing temperature. The increase ranges from 1 to 2%. An increase in drying temperature causes an increase in the brittleness of the material. This is revealed by an increase in the proportion of fine fractions. In the case of the C:1.0 fraction, these temperature levels do not change its proportion. In the case of the C:1.4 fraction, as the temperature increases from 60 to 140 °C, its share decreases by about 4%. This again confirms that higher temperature causes changes in the structure of the material, making it easier to grind. The C:2.0 fraction accounts for a negligible share (less than 1%). Noticeable changes are seen for the 180 and 220 °C levels. The share of the C:0.1 fraction doubles compared to 140 °C (from 4.9% to 10.4% for 180 °C and 11.8% for 220 °C). For the C:0.25; 0.5; and 0.75 fractions, the course of change is similar, but the increments are smaller. The C:1 fraction, stable at about 41% in the 60–140 °C range, dropped to 31% at 180 °C and as low as 28% at 220 °C. Similar trends are found for fraction C:1.4. It can be concluded that the increase in drying temperature to 180 °C causes a distinct change in the material (increase in brittleness) than the range of 60–140 °C. A further increase to 220 °C no longer caused such a significant change.

**Figure 6:**
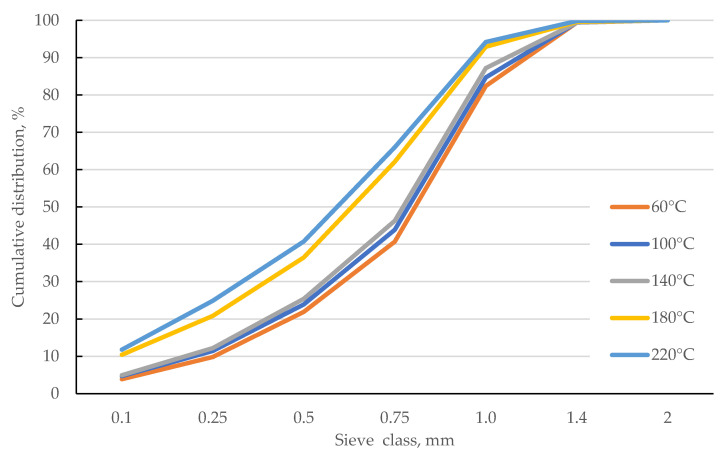
The samples’ cumulative particle size distribution (cPSD).

Figure 6 shows the cumulative PSD (cPSD) for the samples tested. The horizontal red and celadon lines indicate threshold levels of d_10_, d_50_, and d_90_. The cross-section with threshold lines indicates the decrease in d_10_, d_50_, and d_90_ as the drying temperature of corn cobs increases. As the temperature increases, the curve shifts to the left. This means that the fragmentation of corn cob samples increases. For the 60–140 °C range, this shift is small. The largest shift occurred between 140 and 180 °C. An increase in temperature to 220 °C again causes a slight shift of the cPSD curve to the left. Thus, the largest change in structure was caused by the transition from 140 to 180 °C. The changes in cPSD indicate that any increase in drying temperature affects noticeable grindability, but the largest changes are caused by a temperature of 180 °C. Similar trends are observed for biomass in the literature [14,47]. Each temperature level causes structural changes in biomass, but the 180 °C level causes the most significant changes in its brittleness and susceptibility to grinding.

This is confirmed by the values of d_10_, d_50_, and d_90_ (Table 4). In the 60–140 °C range, d_50_ decreases from 0.81 to 0.78 mm, while at the temperature of 180 °C, d_50_ further decreases to 0.63 mm. For 220 °C, d_50_ was 0.59 mm; so, compared to 180 °C, the recorded decrease is small. Similar trends were observed for d_90_. The largest changes were observed for d_10_: in the range of 60–140 °C, the value of d_10_ is in the range of 0.25–0.2 mm. Higher temperature causes d_10_ to drop below 0.1 mm, which means that 10% of the material’s mass is the finest fraction.

### 3.3. Corn Cobs Compressibility and Compactability

Table 5 shows the specific density values of the produced pellets. All samples are characterized by DE above 1 g/cm^3^. ISO 17225-2 and ISO 17225-6 quality standards, for most quality grades, require a pellet bulk density BD ≥ 600 kg/m^3^, in the case of class B non-wood pellets BD ≥ 550 kg/m^3^. Taking into account the shape of the pellet and the associated filling factor of the bed, in order for the pellet to achieve the required BD value, its specific density DE must be above the mentioned 1 g/cm^3^. The DE values obtained are in the range of 1.03–1.17 g/cm^3^. The highest value was obtained for material dried at 180 °C. Regardless of the PSD of the material obtained by milling and the drying temperature, the DE of the pellet remains at a similar level. The pressure used allows the compressibility required by the standards.

**Figure 7:**
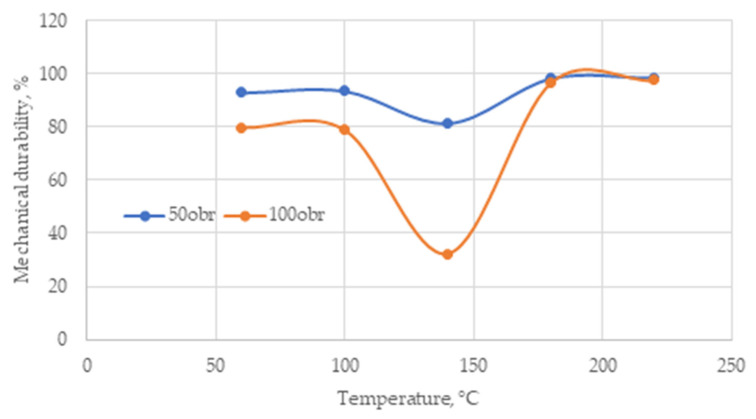
Course of DU changes in corn cobs samples along with increasing drying temperature.

Figure 7 shows the variation in mechanical durability (DU). For the test at 50 turns, pellets from material dried at 60 and 100 °C achieve similar DU of 92.9 and 93.5%, respectively. Increasing the temperature to 140 °C causes the DU to drop to 81.4%. A further increase in temperature to 180 °C causes the DU to rise to 98.4% and remains at a similar level for 220 °C (98.6%). Thus, the least durable pellet with a test count of only 50 turns is the one made from material dried at 140 °C.

An increase in the number of turns (to 100 turns) in the test highlighted this phenomenon even more clearly. The DU of the pellet made from material dried at 140 °C dropped sharply from 81.4% to just 32.3%. Such a value completely disqualifies such a pellet qualitatively. The DU of 60 and 100 °C pellets also dropped from 92.9 to 79.8% and from 93.5 to 78.8%, respectively, which is also not a satisfactory result. In the case of 180 and 220 °C pellets, their DU practically remained at the same level (decreasing from 98.4 to 96.7% and from 98.6 to 97.5%). This is interesting, because despite the doubling of the number of turns of the test, i.e., a doubling of the destructive factor of the pellet, their DU practically did not change. Drying the material at 140 °C did not deteriorate the pellet’s DE; however, it clearly affected the particles’ ability to form permanent bonds during compaction. The grain composition of this material was similar to that dried at 60 and 100 °C, so it was the temperature that changed the structure of the material so much that it does not allow the formation of a durable pellet. In the case of 180 and 220 °C, we observe its positive effect on both DE and DU. The pellets have the highest durability (and density in the case of 180 °C). In this case, the temperature caused the activation of the particles’ properties to form permanent bridges between them. They are so durable that increasing the number of test rotations from 50 to 100 causes virtually no change in DU. In addition, a positive effect on DU of the degree of fineness of the material can also be seen here (a clear shift to the left of the sPSD curves caused by an increase in the proportion of fine fractions, especially C:0.1). Fine particles fit together more easily and their greater number translates into a greater number of bridges that can be formed between them, which directly translates into the mechanical strength of the pellet.

**Figure 8:**
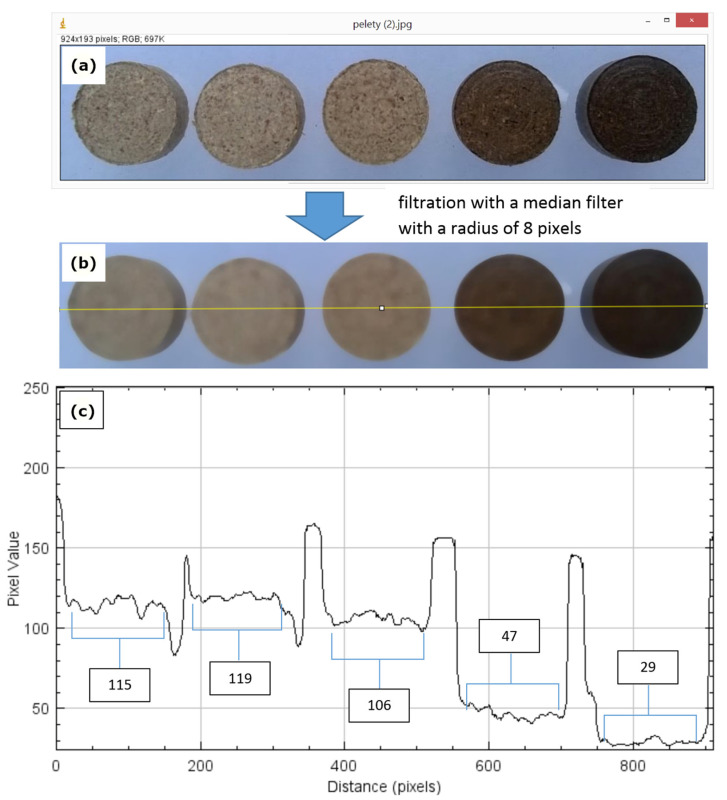
Image processing in program ImageJ: (**a**) baseline image of the research pellet surface, (**b**) image after median filter filtration, (**c**) graph of the intensities of pellet pixels along a line.

To confirm the fact that temperature changes the properties of the material, an analysis of the color changes of the dried material can be used. Pellets made from material dried at 60, 100, and 140 °C clearly differ in color from those from material dried at 180 and 220 °C (Figure 8a).

The exact differences can be visualized using computer image analysis tools. It was realized in a public domain Java image processing program—ImageJ 1.54p [48]. Differences in the brightness of the surface of the pellets were determined. The base image was filtered with a median filter with a radius of 8 pixels to eliminate noise from the input image without significantly blurring it (Figure 8b). Then, a two-dimensional graph of the intensities of pixels along a line was made—a yellow line on the graph (Figure 8c). Based on the obtained values of pixels belonging to the pellet surface, their average value was determined. The drying temperature of 60 and 100 °C results in an average pixel value of 115 and 119, respectively; in this case (lighting conditions, etc.), for the temperature of 140 °C, a decrease to the level of 106 was noted. This is not a significant decrease in terms of the color of the sample; however, the DU results significantly affect the mechanical strength of the pellets. A clear difference is noticeable at 180 and 220 °C, where the pixel value drops to 47 and 29.

The ISO 17225-2 and ISO 17225-6 quality standards, depending on the quality class, require DUs in the range of 98–96% (class A1—98%; A2—97.5%; B—96.5%; and A—97.5% and B—96%). The results obtained, of course, cannot compare to these requirements due to differences in the procedures for implementing strength tests. In the case studied, the test was implemented on single pellets, while the test, according to which we determine the DU of pellets, requires a 500 g sample and a corresponding tester design (ISO 17831-2:2015). The demonstrated differences in DU values nevertheless confirm the influence of drying temperature on this parameter.

The results confirmed the effect of drying temperature on all the tested parameters of the corn cobs samples and the pellets made from them. Table 6 indicates which parameters are required by quality standards and to what extent they are met (*A_d_*, $q_{p,net}$, ar and *DE*). In addition, the values of all parameters have been divided into three groups. The green color indicates the best values of a given parameter (e.g., for *A_d_*, it is its lowest content, and for $q_{p,net}$, it is highest). Correspondingly, the orange color denotes the worst values and the yellow color denotes intermediate values.

Regardless of the drying temperature, the quality parameters required by the ISO 17225-6 standard are met for each drying temperature as Class A. In the case of Class B, after considering the maximum allowable moisture content of *M15%*, the material dried at 60, 180, and 220 °C achieves the required minimum $q_{p,net}$, ar. In the case of ash content, results compared to the ISO 17225-2 standard (wood pellets) allow us to conclude that its requirements are also met to some extent. This only indicates the potential of corn cobs, which, in terms of ash content, meets the requirements for non-wood pellets by a wide margin and even meets class B wood pellets (except for samples dried at 220 °C. However, if we compare $q_{p,net}$, to this standard, it turns out that only material dried at 220 °C meets the requirements. Thus, it should be concluded that, in general, the tested material does not meet the quality classes of ISO 17225-2.

A schematic summary of the results obtained allows us to conclude that 180 °C is the optimal drying temperature. The material meets the requirements of the non-wood pellet standard. It is characterized by satisfactory energy parameters and, in addition, achieves a high degree of fineness. Pellets made from it obtained the highest *DE* and *DU* values. At the same time, it should be stated that corn cob material should not be dried at 140 °C. The ash content is higher and *VM*, $q_{p,net,ar}$ *d_50_* and *DE* are comparable to material dried at 60 and 100 °C. The *DU* of the pellets, on the other hand, is the lowest here.

## 4. Conclusions

The presented results call for further research into the structural composition and the effect of drying temperature on this composition. This will help explain why temperatures of 180 and 220 °C improve grindability (which is consistent with literature data) and why it improves compactibility (which is not confirmed in the literature, but also there are no studies on these temperature values).

Based on the presented test results, the following conclusions can be made:

- Ash content increases with temperature, which is an undesirable but expected phenomenon and consistent with the results available in the literature.
- Volatile content and calorific value increase, which is a desirable phenomenon, and is also confirmed in the literature.
- The value of *d*_50_ decreases, which indicates an increase in grindability. An increase in grindability improves the pressure agglomeration process [49,50,51]. The results obtained also indicate this.
- In the case of specific density, the obtained values are close to each other, so the drying temperature has no significant effect on this parameter.
- The most interesting relationship was observed for the mechanical durability of the pellets. The temperature of 140 °C negatively affected this parameter. According to the literature, roasting worsens compactibility [52]. In the case studied, temperatures of 180 and 220 °C caused the pellets to obtain the highest DU values. The literature states that material dried and actually roasted at 300 °C is more difficult to compact than material roasted at 250 °C. However, the quality of pellets made from steam-exploded Douglas fir has elasticity and higher mechanical strength than those from raw biomass [53]. In our case, the temperature was lower than the typical torrefaction temperature, and perhaps this is where the differences came from. The temperature range studied does not yet result in the decomposition of lignin, which is the main binder during compaction [54].
- The drying of the material in the presented temperature range not only improves its energy parameters (an increase in $q_{p,net}$ and a decrease in volatile parts), but leads to an improvement in the pro-agglomeration properties of the material. In addition, the temperature range studied does not yet require the use of reactors that protect the material from oxygen and thus prevent ignition of the material during roasting.
- Since the presented research has a lab-scale character, further research is required that allows verification of the results on a technical scale. This will allow us to determine the further direction of research on this topic.

## Figures and Tables

**Table 1:** Sieve classes of material samples after preliminary grinding.

Sieve Classes (mm)	B1:0.25	B2:0.5	B3:1	B4:1.4	B5:2	B6:3.15	B7:6	B8:+6
Particle diameter d (mm)	d ≤ 0.25	0.25 < d ≤ 0.5	0.5 < d ≤ 1	1 < d ≤ 1.4	1.4 < d ≤ 2	2 < d ≤ 3.15	3.15 < d ≤ 6	d > 6

**Table 2:** Standardized particle size distribution of the samples.

Sieve Classes (mm)	B_3_:1	B_4_:1.4	B_5_:2
Share (%)	43	26	31

**Table 3:** Sieve classes of material samples after main grinding.

Sieve Classes (mm)	C_1_:0.1	C_2_:0.25	C_3_:0.5	C_4_:0.71	C_5_:1	C_6_:1.4	C_7_:2
Particle diameter d (mm)	d ≤ 0.25	0.25 < d ≤ 0.5	0.5 < d ≤ 1	1 < d ≤ 1.4	1.4 < d ≤ 2	2 < d ≤ 3.15	3.15 < d ≤ 6

**Table 4:** d_10_, d_50_, and d_90_ values of the samples.

Temperature (°C)	60	100	140	180	220
*d*_10_ (mm)	0.25	0.22	0.2	<0.1	<0.1
*d*_50_ (mm)	0.81	0.78	0.78	0.63	0.59
*d*_90_ (mm)	1.18	1.14	1.09	0.98	0.96

**Table 5:** DE values of the samples.

Temperature (°C)	60	100	140	180	220	220
*DE* (g/cm^3^)	1.03	1.03	1.04	1.17	1.07	1.07

**Table 6:** Classification of test results (green—best result, orange—worst result, yellow—intermediate result.

Parameter	Temperature [°C]					ISO Quality Norm
	60	100	140	180	220	
*A_d_*	class B				off-class	17225-2
	all classes					17225-6
						
*VM*						
*q_p_, net at M10%*	off-class				all classes	17225-2
*q_p_, net at M12%*	class A					17225-6
*q_p_, net at M15%*	class B	off-class			class B	17225-6
						
*d50*						
*DE*	all classes					17225-2; 17225-6
						
DU						

## Data Availability

The raw data supporting the conclusions of this article will be made available by the authors upon request.
